# Cosmetic Injectable Treatments Improve Quality of Life in Patients With Multiple Serious Medical Comorbidities

**DOI:** 10.7759/cureus.47458

**Published:** 2023-10-22

**Authors:** Peter Rullan, Kachiu Lee

**Affiliations:** 1 Dermatology, University of San Diego, San Diego, USA; 2 Dermatology, Temple University, Philadelphia, USA

**Keywords:** neuromodulators, co-morbidities, quality of life, cosmetic dermatology, fillers, facial rejuvenation

## Abstract

Full facial rejuvenation with multi-modal cosmetic treatments can improve quality of life, leading to improvement in confidence and psychological function. These combination treatments are frequently administered at the same or sequential sessions and include neuromodulators and fillers. In patients with significant recent weight loss and other medical comorbidities, these treatments can help restore self-confidence and provide the encouragement they need to continue treatment for their comorbidities. We present a case report of a 71-year-old woman who experienced significant weight loss of 40 lbs (33% body weight) during the course of treatment of multiple medical comorbidities, including surgical intervention for compression fractures. Due to her facial appearance caused by this excessive weight loss, she experienced poor self-esteem as measured by the FACE-Q scales. She was treated cosmetically with 21cc of hyperdilute calcium hydroxyapatite (diluted in a 1:3 ratio; 7cc of calcium hydroxyapatite total) and neuromodulators to replace lost facial volume over three visits. At three months after her last treatment, her FACE-Q scores improved two-and-a-half-fold and fivefold on the psychological function and aging appraisal, respectively. Cosmetic treatments can dramatically improve the quality of life in patients with multiple medical co-morbidities. This population of patients is often excluded from clinical trials or other studies, representing a population for which we have little data on the efficacy of cosmetic treatments.

## Introduction

The use of cosmetic treatments, such as neuromodulators and fillers, has been on the rise, with many people selecting to pursue minimally invasive facial rejuvenation procedures due to the perception of their facial appearance [1,2]. Compared to younger patients, older patients are more likely to seek soft-tissue fillers and treatments to tighten facial areas with laxity [3]. Self-described patient motivations for seeking cosmetic treatments include an expectation of improved psychological and personal well-being after treatment [4].

Despite clinical research into improved quality of life after full facial rejuvenation, few of these studies have been conducted on older patients with multiple medical comorbidities. We present a case report of an elderly patient with multiple medical comorbidities causing excessive weight loss, especially in the face.

## Case presentation

A 71-year-old woman presented for facial rejuvenation after losing 40 lbs (33% body weight) over five months due to an array of medical issues and surgical complications. Her past medical history was significant for hyperthyroidism. During that time frame, she also suffered several compression thoracic spine fractures due to her multiple myeloma and osteoporosis. She underwent kyphoplasty to relieve pain but had a hematoma as a surgical complication, which resulted in her being unable to stand up straight, keep food down, or walk and eventually getting pneumonia. She was started on morphine for pain control and placed in a nursing home for five months. The morphine caused severe nausea as well. After five months, her medical comorbidities stabilized, and she was taken off morphine. She returned home with no further medical or surgical treatment plans and noted that her physicians had “lost hope” and advised her to avoid further medical treatments for her multiple myeloma given the recent complications from her surgical treatments.

**Figure 1:**
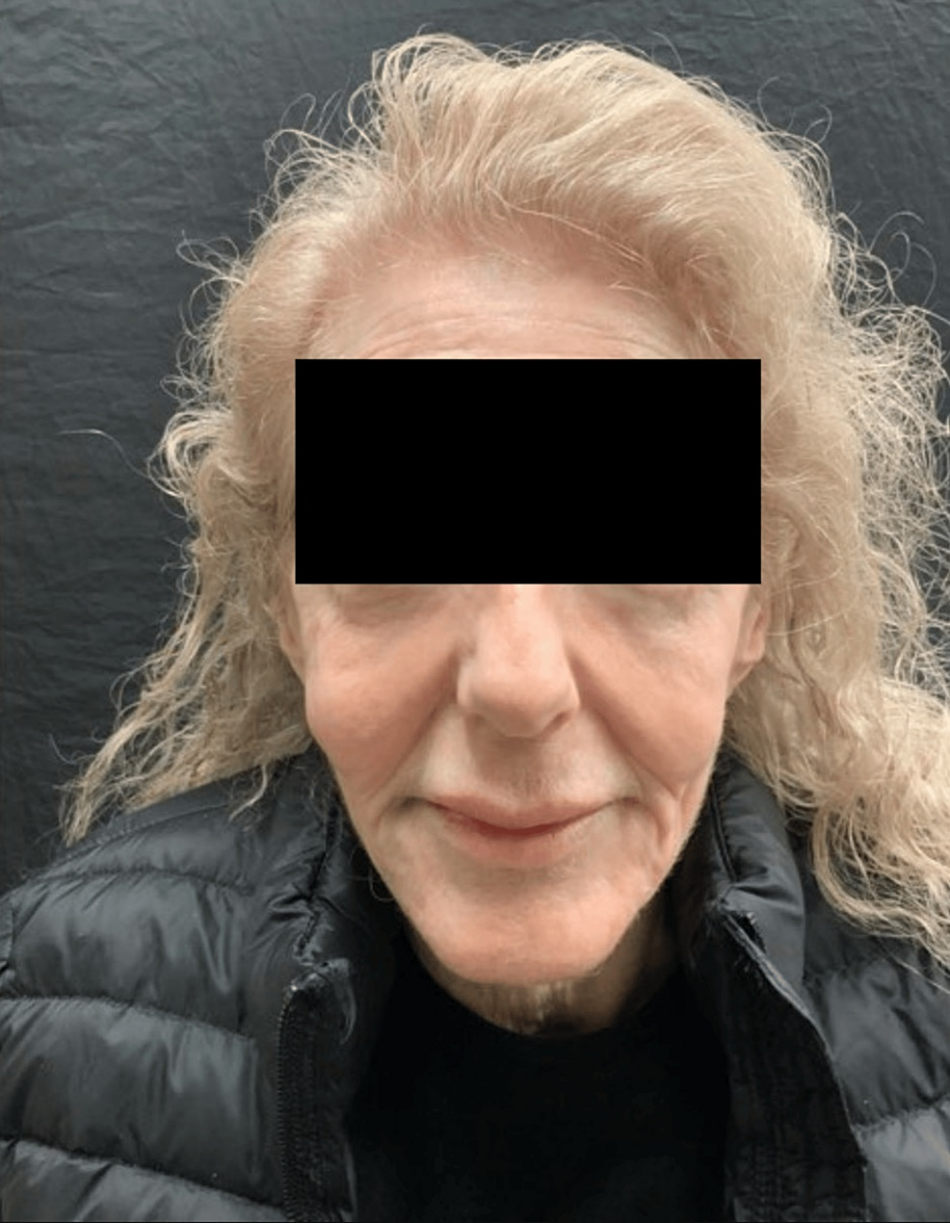
71-year-old woman at baseline prior to any cosmetic injectable treatments

On examination, the patient had a BMI of 17.4 with a cachectic and gaunt appearance (Figure 1).

**Figure 2:**
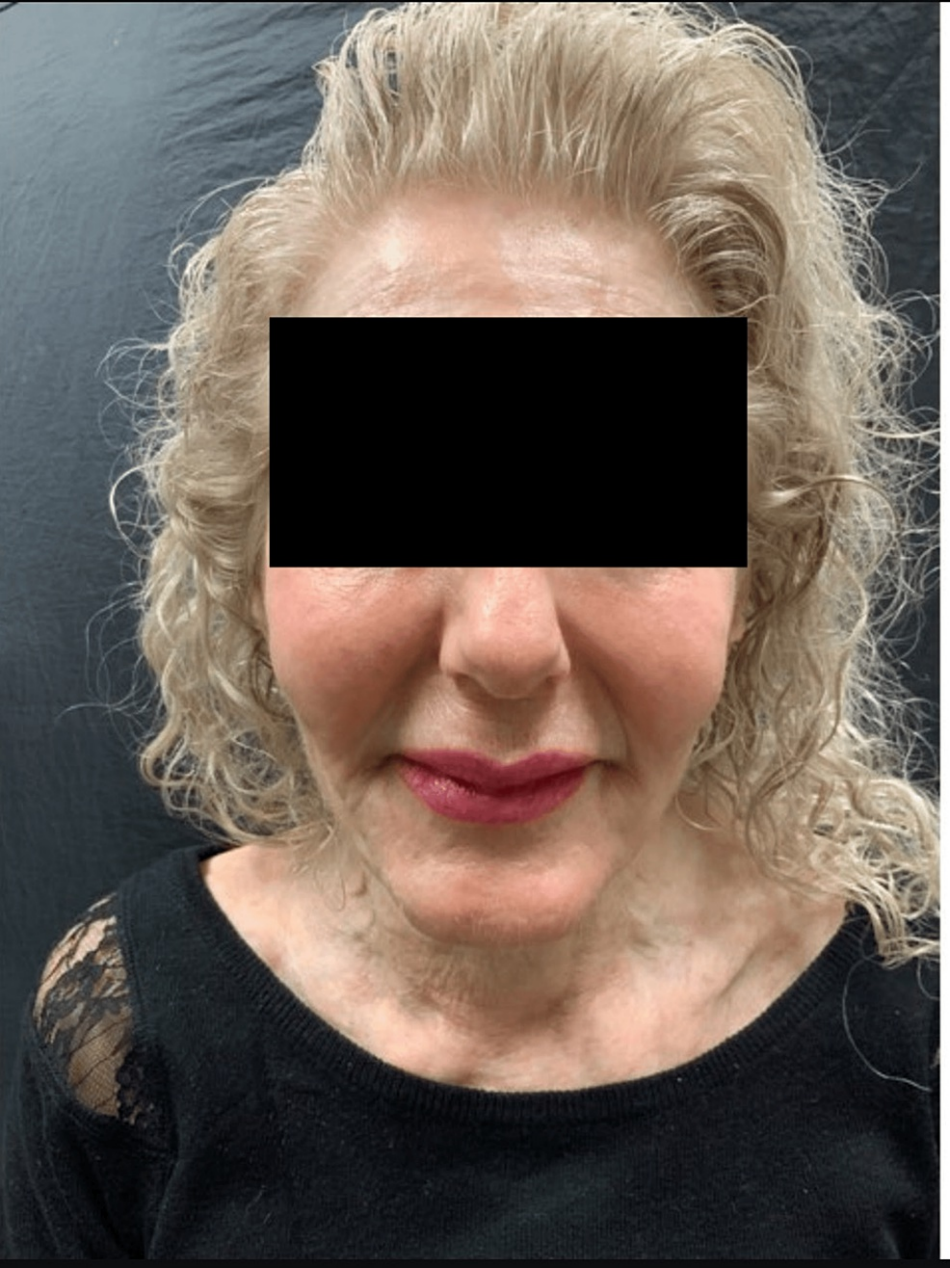
71-year-old woman after three calcium hydroxyapatite treatments to the cheeks, jawline, and nasolabial folds

She maintained that she was actively trying to regain weight but felt distressed by her current appearance. After a thorough discussion about treatment options, the patient opted for volumizing fillers and neuromodulators. We injected calcium hydroxyapatite (CaHA) diluted one-to-one with sodium chloride, for a total of 21 cc (seven syringes of CaHA) over three monthly visits, using a 22 gauge, two-inch cannula. Areas treated included the cheeks, jawline, and nasolabial folds (Figure 2).

To assess her quality of life after this full facial multi-modality treatment, the patient filled out baseline and post-treatment FACE-Q aging appraisal, appearance-related psychological distress, and psychological function surveys. Post-treatment surveys were administered three months after the last treatment. All FACE-Q surveys showed improvement post-treatment, with the aging appraisal improving over fivefold (baseline score of 15; three-months post-treatment score of 83), appearance-related psychological distress improving over threefold (baseline score of 31; three-months post-treatment score of 100), and psychological function improving over two and a half-fold (baseline score of 31; three-months post-treatment score of 82).

The patient also shared that both her primary doctor and her orthopedic surgeon were previously only offering palliative care until they saw her improved recent cosmetic appearance. They have now become more optimistic about her chances for recovery and are planning to do an MRI and possibly another kyphoplasty. Her thyroid disease is also under better control since starting medication, and her multiple myeloma is stable. She has gained 8-9 lbs in the last three months.

## Discussion

We present a case of a 71-year-old woman who achieved remarkable two-and-a-half-fold to three-fold improvement in her FACE-Q scores after treatment with injectables, specifically CaHA, and neuromodulators. Facial rejuvenation procedures are increasing in popularity, with over $15 billion spent on cosmetic procedures in 2021 [5].

No studies have evaluated the quality of life in patients with multiple comorbidities that may cause dramatic weight loss or loss of facial volume. Therefore, there are no existing studies or case reports that we can compare with our current results. This population comprising people with active and serious medical illnesses requiring interventions, such as surgery, chemotherapy, or rehabilitation, has not been previously studied.

Our case report showed that facial rejuvenation treatments can dramatically improve quality of life, boost self-esteem, and motivate patients. What is notable about our case study is that, despite her chronic and serious ongoing medical issues, we opted to treat this patient with the goal of improving her quality of life. Compared to previously published studies on quality of life after full facial rejuvenation, our patient had much lower FACE-Q scores at baseline, which is perhaps reflective of the multiple comorbidities and the influence it had on her personal outlook [6-9].

## Conclusions

Oftentimes, patients with active cancer or other serious illnesses are excluded from clinical trials or other studies due to their medical history and are, thus, underrepresented in our medical literature. We present a case report of a patient with dramatic weight loss due to medical comorbidities with incredibly low FACE-Q scores reflecting her self-esteem and poor outlook. After full facial rejuvenation with fillers, her FACE-Q scores improved dramatically. This case illustrates how cosmetic treatments can dramatically improve a patient’s quality of life despite dealing with multiple medical comorbidities and limitations.
